# PACmn for improved optogenetic control of intracellular cAMP

**DOI:** 10.1186/s12915-021-01151-9

**Published:** 2021-10-18

**Authors:** Shang Yang, Oana M. Constantin, Divya Sachidanandan, Hannes Hofmann, Tobias C. Kunz, Vera Kozjak-Pavlovic, Thomas G. Oertner, Georg Nagel, Robert J. Kittel, Christine E. Gee, Shiqiang Gao

**Affiliations:** 1 Department of Neurophysiology, Institute of Physiology, Biocenter, Julius-Maximilians-University of Würzburg, Röntgenring 9, 97070 Würzburg, Germany; 2 Institute for Synaptic Physiology, Center for Molecular Neurobiology Hamburg, University Medical Center Hamburg-Eppendorf, 20251 Hamburg, Germany; 3 Department of Animal Physiology, Institute of Biology, Leipzig University, Talstraße 33, 04103 Leipzig, Germany; 4 Carl-Ludwig-Institute for Physiology, Leipzig University, Liebigstraße 27, 04103 Leipzig, Germany; 5 Department of Microbiology, Biocenter, Julius-Maximilians-University of Würzburg, Am Hubland, 97074 Würzburg, Germany

**Keywords:** Second messenger, Cyclic AMP, bPAC, Photoactivatable adenylyl cyclase, *Xenopus* oocyte, *Drosophila*, Rat, Neuron

## Abstract

**Background:**

Cyclic adenosine monophosphate (cAMP) is a ubiquitous second messenger that transduces extracellular signals in virtually all eukaryotic cells. The soluble *Beggiatoa* photoactivatable adenylyl cyclase (bPAC) rapidly raises cAMP in blue light and has been used to study cAMP signaling pathways cell-autonomously. But low activity in the dark might raise resting cAMP in cells expressing bPAC, and most eukaryotic cyclases are membrane-targeted rather than soluble. Our aim was to engineer a plasma membrane-anchored PAC with no dark activity (i.e., no cAMP accumulation in the dark) that rapidly increases cAMP when illuminated.

**Results:**

Using a streamlined method based on expression in *Xenopus* oocytes, we compared natural PACs and confirmed bPAC as the best starting point for protein engineering efforts. We identified several modifications that reduce bPAC dark activity. Mutating a phenylalanine to tyrosine at residue 198 substantially decreased dark cyclase activity, which increased 7000-fold when illuminated. Whereas *Drosophila* larvae expressing bPAC in mechanosensory neurons show nocifensive-like behavior even in the dark, larvae expressing improved soluble (e.g., bPAC(R278A)) and membrane-anchored PACs exhibited nocifensive responses only when illuminated. The plasma membrane-anchored PAC (PACmn) had an undetectable dark activity which increased >4000-fold in the light. PACmn does not raise resting cAMP nor, when expressed in hippocampal neurons, affect cAMP-dependent kinase (PKA) activity in the dark, but rapidly and reversibly increases cAMP and PKA activity in the soma and dendrites upon illumination. The peak responses to brief (2 s) light flashes exceed the responses to forskolin-induced activation of endogenous cyclases and return to baseline within seconds (cAMP) or ~10 min (PKA).

**Conclusions:**

PACmn is a valuable optogenetic tool for precise cell-autonomous and transient stimulation of cAMP signaling pathways in diverse cell types.

**Supplementary Information:**

The online version contains supplementary material available at 10.1186/s12915-021-01151-9.

## Background

Since its discovery as the first second messenger, 3’,5’-cyclic adenosine monophosphate (cAMP) has become known as an indispensable signaling molecule in virtually all cells. Adenylyl cyclases cyclize adenosine triphosphate (ATP) to form cAMP [1], which activates downstream effectors including the cyclic nucleotide-gated (CNG) [2] and hyperpolarization-activated cyclic nucleotide-gated (HCN) ion channels [3], cAMP-dependent protein kinase A (PKA) [4], and exchange protein activated by cAMP (EPAC) [5]. In animals, most adenylyl cyclases (AC1-9) are membrane proteins activated downstream of metabotropic receptors or by intracellular Ca^2+^ [6, 7]. A soluble adenylyl cyclase (sAC or AC10) is activated by bicarbonate and plays important roles in sperm motility and fertilization [8–10].

Optogenetic manipulation of cAMP was first achieved by expressing the photoactivated adenylyl cyclase from the unicellular alga *Euglena gracilis* (EuPAC) in *Xenopus* oocytes, in HEK293 cells, and in *Drosophila melanogaster*, where neuronal expression yielded light-induced changes in behavior [11, 12]. However, the large size, low solubility, and significant dark activity hindered the widespread application of EuPAC. The discovery of a small PAC from the soil bacterium *Beggiatoa* (bPAC) with low dark activity and high light activity provided a versatile tool for manipulating and studying cAMP-mediated processes in individual, genetically targeted cells [13, 14]. Applications include rescue of infertility, controlling insulin release, neuronal repair, and modulating release of neurotransmitters and synaptic plasticity [15–21]. Compared to pharmacological manipulation, the rapid reversibility and increased temporal and spatial resolution conferred by using bPAC is an advantage when studying rapid and compartmentalized cAMP signaling [22–25]. Due to its smaller size, bPAC is more amenable for subcellular targeting than other optogenetic tools such as CaRhAC [26] derived from the enzyme rhodopsin CaRhGC (CaCyclOp) [27, 28]. For example, bPAC has been targeted to presynaptic terminals (SynaptoPAC) revealing a cAMP-dependent increase in evoked transmission at hippocampal mossy fibers but not at CA3-CA1 synapses [29].

While bPAC is a valuable tool, it has some drawbacks. Though the dark activity is low, bPAC expression increases resting cAMP in some cells. Also, bPAC is a soluble protein, raising cAMP throughout the cytoplasm whereas most eukaryotic adenylyl cyclases are transmembrane proteins. A PAC targeted to the plasma membrane that does not raise basal cAMP in the dark is therefore highly desired.

Here, we present a simple and sensitive method using the *Xenopus* oocyte expression system for characterizing the enzymatic activity of soluble and membrane cyclases. Using this assay, we systematically characterized and compared natural PACs including bPAC [13, 14], EuPAC [12], TpPAC [30], mPAC [31], and LiPAC [32]. The superior candidate bPAC was further mutated and fused with membrane anchors to generate variants with reduced dark activity, a high light-dark ratio, and faster kinetics. Unlike wild-type bPAC, the selected constructs only induce nocifensive behavior upon illumination of *Drosophila* larvae and not in the dark. PACmn (PAC membrane-anchored, no dark activity, pronounced PAC-man), neither increases cAMP nor increases resting cAMP-dependent protein kinase (PKA) activity in the dark. When illuminated, cyclase activity increases > 4000-fold activating ion channels, PKA, and presumably other cAMP-dependent downstream signaling pathways.

## Results

### The low dark activity of bPAC overcomes phosphodiesterases to raise cAMP in *Xenopus* oocytes and hippocampal neurons

**Fig. 1:**
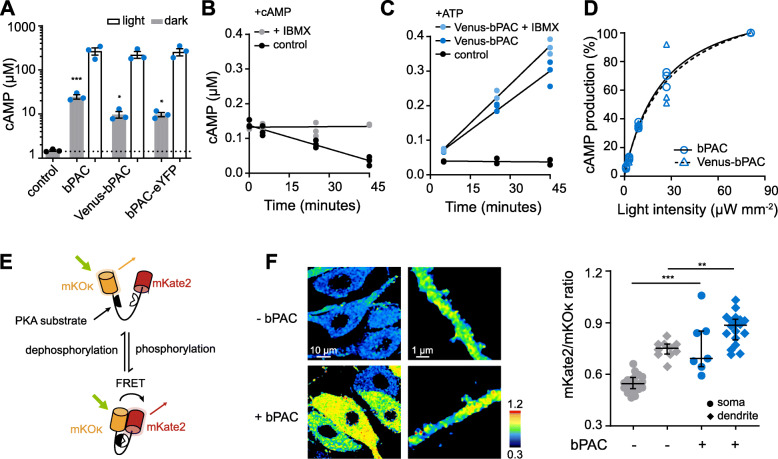
bPAC light and dark activity. **A** cAMP concentrations from whole *Xenopus* oocytes kept in the dark (dark) or after illumination with 0.3 mW mm^-2^ 473 nm for 30 s (light). Data are mean ± SEM, *n* = 3 experiments with 5 oocytes each. ***p < 0.0001, *p < 0.05, Dunnett’s multiple comparisons vs control following one-way ANOVA (*p* < 0.0001). **B** Endogenous PDE activity in oocyte extracts. At time 0 minutes 0.15 μM cAMP was added to the soluble extracts in the absence (control) or presence of 1 mM IBMX (+ IBMX). *n* = 3 experiments, extracts pooled from 15 oocytes. **C** cAMP production of Venus-bPAC containing soluble extract after adding ATP into the reaction buffer in the absence and presence of 1 mM IBMX under dark condition. Note that the control oocyte (black in **C**) did not produce any cAMP after adding ATP. **D** Light (473 nm) intensity dependence of mean normalized cAMP production by Venus-bPAC or bPAC at 473 nm. *K*_m_ = 26.2 μW mm^-2^ and 24.8 μW mm^-2^, respectively. **E** Working mechanism of the PKA activity FRET sensor Booster-PKA. **F** Representative ratio images (mKate2/mKoκ) of the hippocampal neurons expressing Booster-PKA alone or together with bPAC. Shown at right are individual measurements, median, and interquartile range. ***p < 0.0001, **p < 0.001, unpaired *t* tests. *n* = 20, 10, 7, and 18

Stage V and VI *Xenopus* oocytes have a cAMP concentration of several μM [28]. The cAMP concentration was, however, 24.7 ± 4.7 μM in oocytes expressing bPAC for 3 days in the dark (vs 1.48 ± 0.1 μM in non-injected oocytes, mean ± SD, Fig. 1A). Adding fluorescent tags to the N (Venus-bPAC) or C terminus (bPAC-eYFP) decreased the dark accumulation of cAMP to 9.7 ± 2.8 μM and 9.7 ± 1.9 μM, respectively, without affecting the light-induced cAMP (Fig. 1A, bPAC 267 ± 65 μM, Venus-bPAC 223 ± 67 μM, bPAC-eYFP 257 ± 83 μM). As the dark activity of bPAC clearly increases cAMP, we set out to engineer or identify naturally occurring PACs that do not alter cAMP concentrations in the dark.

To facilitate characterizing the enzymatic properties of soluble and membrane adenylyl cyclases expressed in *Xenopus* oocytes, we optimized previously published methods using crude membrane and soluble cytoplasmic extracts together with fluorescence-based quantification [28, 33, 34]. Ultrafiltration removed small proteins and small molecules (e.g., ATP/GTP, cAMP/cGMP, Mg^2+^, and Ca^2+^), which might influence the determination of nucleotidyl cyclase activity from cytoplasmic extracts. However, large proteins such as endogenous phosphodiesterases (PDEs) and sACs remain after ultrafiltration. To quantify endogenous PDE activity, 0.15 μM cAMP was added to cytoplasmic extracts in the presence and absence of 1 mM IBMX (a PDE inhibitor). While cAMP concentrations were constant in the presence of 1 mM IBMX, degradation of cAMP was observed in control extracts, clear evidence of endogenous PDE activity (Fig. 1B). In contrast, no cAMP was produced in soluble extracts from control oocytes after adding 1 mM ATP (Fig. 1C, 0.036 ± 0.011 μM), consistent with the extremely low activity of endogenous sACs [35]. In extracts from Venus-bPAC expressing oocytes, the cAMP concentration increased in the dark to 0.30 ± 0.04 μM and 0.37 ± 0.02 μM in the absence and presence of IBMX, respectively (Fig. 1C). Therefore, to accurately determine dark cyclase activity, IBMX was always included to block endogenous PDEs. However, adding IBMX did not change light-induced Venus-bPAC cAMP production, indicating that the light activity overwhelms the endogenous PDEs (12.6 ± 0.3 μM vs 13.3 ± 0.6 μM with IBMX, *p* = 0.12, *n* = 3, 4 min, 473 nm, 0.3 mW mm^-2^). Using the same method, we verified that light-induced bPAC activity was not affected by the N terminal Venus tag (Fig. 1D).

One of the main downstream effectors of cAMP is PKA. To test whether bPAC also increases resting cAMP in the hippocampal neurons, we used the orange-red Förster resonance energy transfer (FRET) sensor Booster-PKA [36]. Upon phosphorylation by PKA, FRET increases, meaning the ratio of the acceptor (mKate2) to donor (mKOκ) fluorescence increases (Fig. 1E). As donor fluorescence can be excited at wavelengths (i.e., 550 nm) longer than are absorbed by the BLUF (blue light receptor using flavin adenine dinucleotide) domain of bPAC (350–520 nm), Booster-PKA imaging is compatible with bPAC. Co-expression of bPAC increased Booster-PKA FRET in the dark (no PDE inhibitors were added), indicating that bPAC increases cAMP and in turn PKA activity in the hippocampal neurons (Fig. 1F). Thus, the dark activity of wild-type bPAC is sufficient to overcome not only the *Xenopus* oocyte PDEs but also the PDEs in the rat hippocampal neurons.

### Of natural soluble photoactivatable adenylyl cyclases bPAC has the highest light to dark ratio

We compared the activities of published natural PACs fused with fluorescent proteins at either N or C termini. The light activity of the tagged bPACs was the highest at around 90 min^-1^ with a light to dark ratio around 1600 and neither light nor dark activity of bPAC(wt) was affected by the position of the fluorescent tags (Table 1). EuPACα-eYFP displayed 6-fold higher dark activity than bPAC(wt)-eYFP and lower light activity, resulting in a L/D ratio of 180 (Table 1). Moving the eYFP to the N terminus of EuPAC led to a 6-fold reduction in light activity of eYFP-EuPAC, while the dark activities remained similar (Table 1). The cyanobacterial mPAC showed 30 times higher dark activity than bPAC(wt) while its light activity was only half of bPAC(wt). No light or dark cyclase activity of LiPAC was detected (Table 1). TpPAC had slightly lower dark activity than bPAC(wt), but unfortunately, its light activity was 18 times lower, generating a L/D ratio of only 150 (Table 1). Of the naturally occurring PACs we tested, bPAC(wt) was superior having the best combination of low dark activity and high light activity so we focused our engineering efforts towards reducing the dark activity of bPAC.

**Table 1:** Enzymatic activity of selected PACs

Construct	Dark turnover (min^**-1**^)	Light turnover (min^**-1**^)	L/D
bPAC(wt)-eYFP	0.057 ± 0.015	93 ± 9	1630
Venus-bPAC(wt)	0.055 ± 0.01	91 ± 8	1650
EuPACα-eYFP	0.38 ± 0.06	67.6 ± 9.1	180
eYFP-EuPACα	0.35 ± 0.08	11.9 ± 1.6	34
mPAC-eYFP	1.55 ± 0.4	43.3 ± 5.5	28
LiPAC-eYFP	< 0.0005	< 0.0005	-
eYFP-LiPAC	< 0.0005	< 0.0005	-
TpPAC-eYFP	0.033 ± 0.006	5 ± 0.6	151
eYFP-TpPAC	0.04 ± 0.007	4 ± 0.4	100
bPAC(K197A)-eYFP	0.0066 ± 0.0006	2.6 ± 0.5	390
bPAC(R278A)-eYFP	0.0012 ± 0.0003	1.6 ± 0.3	1330
Venus-bPAC(S27A)	0.023 ± 0.004	94.3 ± 5.9	4100
Venus-bPAC(F198Y)	≤ 0.0024	17 ± 0.8	7080
CD8-Venus-bPAC(wt)	0.011 ± 0.0015	16.8 ± 2.0	1530
CD8-Venus-bPAC(R278A)	< 0.0005	0.52 ± 0.17	> 1000
Glyco-Venus-bPAC(wt)	0.0076 ± 0.0012	17.5 ± 3.4	2300
Glyco-Venus-bPAC(S27A)	0.0051 ± 0.0002	15 ± 0.6	2930
Glyco-Venus-bPAC(R278A)	< 0.0005	0.51 ± 0.05	> 1000
Lyn-Venus-bPAC(wt)	0.014 ± 0.003	18.2 ± 2.3	1300
2xLyn-Venus-bPAC(wt)	0.013 ± 0.003	16.9 ± 2.4	1300
2xLyn-Venus-bPAC(S27A)	0.0067 ± 0.002	18.3 ± 2.5	2730
*2xLyn-Venus-bPAC(F198Y)	< 0.0005	2 ± 0.3	> 4000

*PACmn

Values are mean ± SD, *n* = 3 experiments, 15 oocytes each experiment (turnover = molecules cAMP produced by 1 molecule PAC in one min)

### Reducing bPAC dark activity by point mutations and membrane targeting

bPAC functions as a dimer formed by the interactions of two BLUF α3 helices. In the dark, bPAC occasionally assumes a catalytically competent conformation and generates cAMP. Light absorption extends the opening angle and re-positions the catalytic residues to better interact with substrate and catalyze the reaction [37]. Accordingly, modifying the interactions between the two BLUF α3 helices might affect the opening angle of two AC domains (e.g., L123). In addition, mutating substrate accommodating related residues (e.g., K197, F198, H266, T267) or catalytically important residues (e.g., R278) could in principle reduce the dark activity of bPAC [37]. Therefore, we introduced point mutations in these residues to reduce bPAC dark activity.

**Fig. 2:**
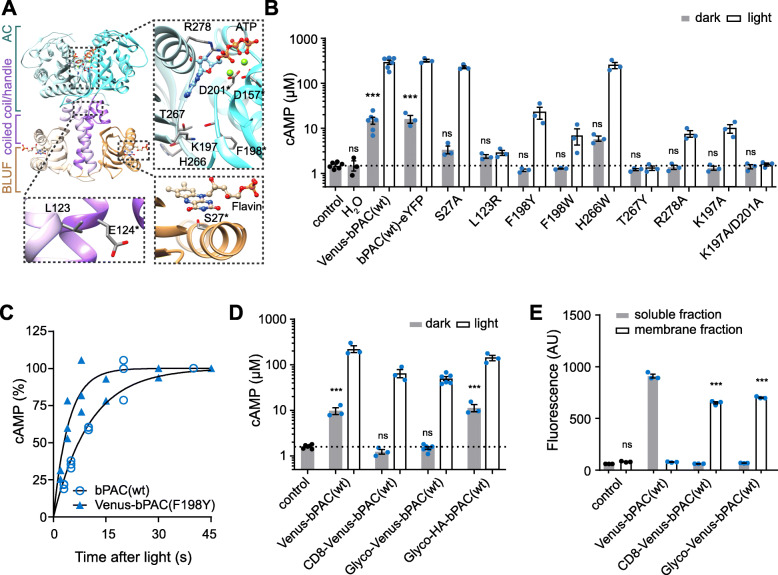
Reducing bPAC dark activity. **A** Model of bPAC structure (5MBK, 5M2A), a parallel homodimer (chain A and B colored in light and dark shades), marked with the “FAD” chromophore, ATP substrate, and residues of interest. Asterisks indicate residues from the other chain of the dimer. Green spheres represent the two catalytic Mg^2+^ ions. **B** Point mutations of key residues and their effect on dark and light cAMP concentration in whole oocytes as in Fig. 1A. All bPAC mutants are either tagged with Venus at the N-terminus (S27A, L123R, F198Y, F198W, H266W, and T267Y) or eYFP at the C-terminus (K197A, K197A/D201A, and R278A). **C** Normalized cAMP production at different time delays in the dark after 500 ms, 473 nm, 0.3 mW mm^-2^ light stimulation fitted with a mono-exponential function (bPAC(wt) τ = 10.1 s, Venus-bPAC(F198Y) *τ* = 4.5 s. **D** Effect of membrane anchoring on cAMP concentration of whole oocytes in dark and light conditions as in Fig. 1A. **E** Cytoplasmic and membrane distribution of bPAC variants indicated by Venus fluorescence. Data in **B**, **D**, and **E** are individual values and mean ± SEM. *n* = 3–6. Control = non-injected oocytes. ***p < 0.0001, *p < 0.05, ns = not significant, Dunnett’s multiple comparisons vs control following one-way ANOVA (*p* < 0.0001)

We first tested S27A, which has been reported to reduce dark activity without changing the light activity [38]. The dark activity was indeed reduced to 40% and resting cAMP in oocytes was no longer significantly increased (Fig. 2B, Table 1). We did not consider this mutation further due to the 15 nm increase in peak absorption, which prevents using it with other green light sensitive optogenetic tools.

We hypothesized that L123R would stabilize the two helices by forming a salt bridge with E124* (* indicates residues of the dimer-partner), but this mutation unfortunately abolished both dark and light activity (Fig. 2A, B). Structural analysis also revealed that residue F198* might affect the accessibility of K197 for ATP binding (Fig. 2A). In the dark state, the side chain of K197 interacts with the carbonyl oxygens of F198* and H266 (Fig. 2A). Light stimulation would free K197 from F198* to allow binding to the substrate ATP. We therefore tested whether mutating H266 and F198 to larger residues might reduce the access of ATP to K197 in the dark and thus reduce dark activity. Indeed, F198Y and F198W reduced the dark activity, but the H266W mutation had little effect (Fig. 2B). The dark turnover of F198Y decreased to about 4% of bPAC(wt) (≤ 0.0024 min^-1^), whereas its light activity was only reduced to 18%, resulting in an L/D ratio above 7000 (Table 1). Besides having the highest L/D ratio, the cyclase activity of F198Y spontaneously returns to the inactive state in the dark 2.2x faster than bPAC(wt). After a light flash of 500 ms, the cAMP concentration increased in the dark reaching a plateau when the enzyme activity stopped (Fig. 2C). F198W had strongly reduced light activity and was not tested further. Mutating another major determinant of base recognition to T267Y abolished both dark and light activity (Fig. 2B). Interestingly, K197A and R278A showed no cAMP accumulation in the dark and were still photoactivatable whereas the double mutant K197A/D201A lost all catalytic activity (Fig. 2C). The dark activities of K197A and R278A were determined to be 0.0066 min^-1^ and 0.0012 min^-1^, respectively, which corresponded to 10% and 2% of the bPAC(wt) dark activity (Table 1). The L/D ratio of K197A was reduced to 400 while R278A was similar to bPAC(wt) (Table 1).

As most eukaryotic PACs are transmembrane proteins, we targeted bPAC to the plasma membrane. We first tested bPAC(wt) fused to CD8 [39] or the first 150 aa of Glycophorin A (Glyco). Interestingly, CD8-Venus-bPAC(wt) and Glyco-Venus-bPAC(wt) expressing oocytes accumulated less cAMP in the dark than Venus-bPAC(wt) and retained a strong light response (Fig. 2D). The length or shape of the linker was important as replacing Venus with a short HA tag increased dark cAMP accumulation (Fig. 2D). We confirmed the membrane localization in oocytes and HeLa229 cells (Fig. 2E, Additional file 1: Figure S1). Whereas CD8-Venus-bPAC(wt) was located mostly in the inner membranes, Glyco-Venus-bPAC(wt) targeted well to the plasma membrane ruffles in HeLa299 cells (Additional file 1: Figure S1). In terms of enzymatic activity, Glyco-Venus-bPAC(wt) showed slightly higher light activity and lower dark activity than CD8-Venus-bPAC(wt) (Table 1). Incorporating the S27A point mutation into Glyco-Venus-bPAC further increased the L/D ratio by decreasing dark activity (Fig. 2A, Table 1), as expected [38].

### Engineered bPAC variants only induce *Drosophila* nocifensive behavior in the light

**Fig. 3:**
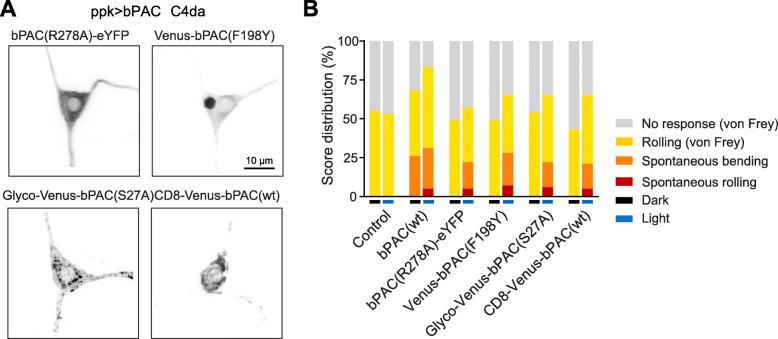
Behavioral assessment of bPAC variants in *Drosophila*. **A** Mechanical nociceptors (C4da sensory neurons) expressing bPACs (grey, *ppk-GAL4* driver). The soluble R278A and F198Y variants and Glyco-Venus-bPAC(S27A) are located in the cell body and dendrites. CD8-Venus-bPAC(wt) is confined to the somatic region of C4da neurons. **B** Nocifensive behavior in groups of larvae expressing bPAC variants in C4da neurons in the dark (black bar) or blue light (blue bar). Note that control larvae exhibited no nocifensive behavior except in response to von Frey filament stimulation and larvae expressing unmodified bPAC showed nocifensive behavior in the dark

We used the bipartite *UAS/GAL4* system to express bPAC variants in larval *Drosophila* neurons and test their performance in vivo. In motoneurons (*ok6-GAL4* driver), the soluble variants bPAC(R278A)-eYFP and Venus-bPAC(F198Y) displayed expression in cell bodies in the ventral nerve cord (VNC), in axons leaving the VNC, and at the neuromuscular junction (NMJ) (Additional file 1: Figure S2). Membrane-targeted Glyco-Venus-bPAC(S27A) also localized throughout the neuron showing a speckled distribution in all compartments. CD8-Venus-bPAC(wt) appeared restricted to cell bodies and did not localize to axons or the NMJ (Additional file 1: Figure S2). C4da neurons are involved in mechanical nociception and express the Degenerin/epithelial sodium channel *pickpocket* (*ppk*) [40, 41]. Driven by *ppk-GAL4*, the soluble bPAC(R278A)-eYFP variant distributed uniformly throughout the cell body and dendrites whereas Venus-bPAC(F198Y) showed an additional structure in the cell body that however did not appear to affect the general morphology or function of these neurons (Fig. 3A, Additional file 1: Figure S3). As in motoneurons, the membrane-targeted Glyco-Venus-bPAC(S27A) was well-distributed but speckled whereas CD8-Venus-bPAC(wt) localized in the soma with little expression in the dendrites (Fig. 3A, Additional file 1: Figure S3).

*Drosophila* larvae show stereotyped behavioral responses to noxious mechanical insult, most notably the “corkscrew” body roll, a rapid rotation along the animal’s long axis, which can be triggered by stimulation with a von Frey filament or direct C4da neuron activation [42, 43]. Recent work showed that optogenetic cAMP production by bPAC sensitizes C4da neurons and promotes nocifensive behavior [44]. We therefore expressed the bPAC variants in C4da neurons (*ppk-GAL4* driver) and quantified nocifensive responses as an in vivo readout for cAMP production. Mechanical stimulation with a 40 mN von Frey filament triggered a nocifensive body roll in 55% of control larvae irrespective of illumination (Fig. 3B). This confirms that the chosen light settings are innocuous. Photostimulation of animals expressing bPAC(wt) elicited nocifensive behavior even without Frey filament stimulation (“spontaneous bending” and “spontaneous rolling”; Fig. 3B), consistent with nociceptor sensitization by cAMP. Notably, the larvae also displayed spontaneous bending in the dark, likely due to the dark activity of bPAC(wt) [13, 44]. This undesirable dark phenotype was removed in larvae expressing the new variants (Fig. 3B). These bPAC versions therefore enable tighter control of the cAMP production.

### In hippocampal neurons PACmn rapidly increases cAMP signaling without effects in the dark

**Fig. 4:**
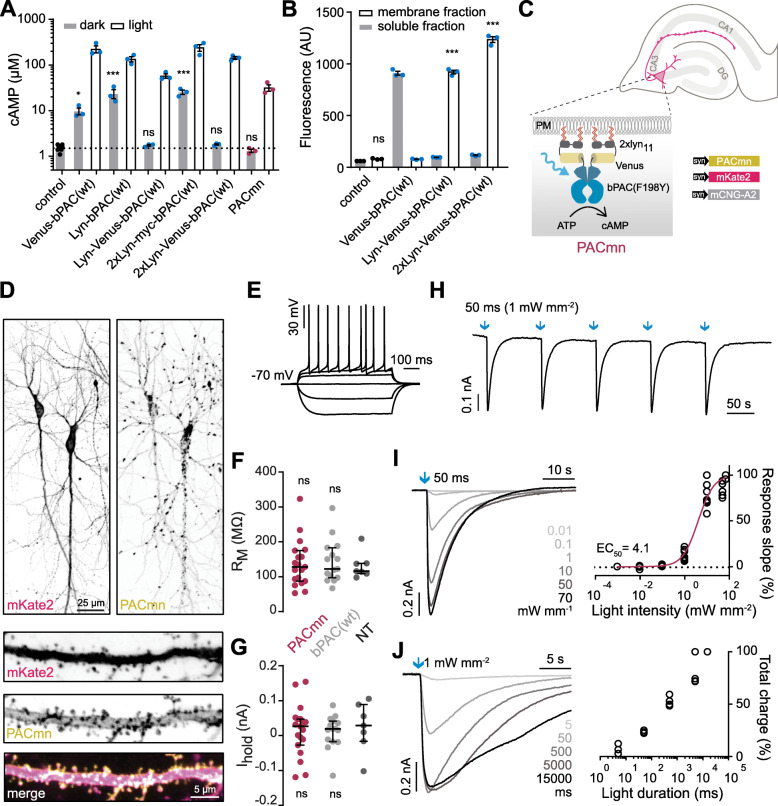
Development of membrane-targeted PACmn and characteristics of the light-evoked responses in expressing hippocampal neurons. **A** cAMP concentrations of whole oocytes expressing soluble or membrane-targeted (Lyn) bPAC variants in dark and light conditions as in Fig. 1A. Mean ± SEM, *n* = 3 experiments with 5 oocytes each. ***p < 0.0001, *p < 0.05, Dunnett’s multiple comparisons vs control. **B** Cytosol and membrane distribution of bPAC variants in oocytes indicated by Venus fluorescence, as in Fig. 2D. **C** PACmn construct design and hippocampal slice culture electroporation strategy. **D** Confocal projection images of hippocampal neurons co-expressing PACmn (2xLyn-Venus-bPAC(F198Y)) and mKate2, together with close-up of an apical dendrite. Merged image PACmn (yellow) mKate2 (magenta) colocalization appears white. **E** Whole-cell responses to somatic current injections from −400 pA to 700 pA in a PACmn expressing neuron in the dark (resting membrane potential −70 mV). **F** Membrane resistance and **G** holding current of non-transfected (NT), PACmn + mCNG, or bPAC(wt) + mCNG—expressing neurons when clamping the membrane voltage at −70 mV. **H** Photocurrents evoked by five consecutive 470 nm light flashes (50 ms, 1 mW mm^-2^, interval 100 s) in a PACmn + mCNG expressing neuron. **I** Representative currents recorded from PACmn and mCNG expressing hippocampal neurons in response to 50 ms 470-nm light pulses of varying intensity and the light intensity-response relationship fitted with a quadratic equation. Currents were normalized to the maximum current recorded for each neuron. **J** Representative currents recorded from PACmn and mCNG expressing hippocampal neurons in response to 1 mW mm^-2^ 470 nm light pulses of varying duration (5 ms to 15 s). At right is the light duration-response relationship (total charge). The maximum charge from each neuron was set to 100%

As expression of the soluble Venus-bPAC(F198Y) and Glyco-Venus-bPAC(wt) was not optimal in hippocampal neurons (Additional file 1: Figure S1), we explored smaller membrane anchor peptides (MGCIKSKGKDS) from the tyrosine kinase Lyn. Lyn-bPAC(wt) (bPAC-PM in ref [45]) increased cAMP in the dark (Fig. 4A). Increasing the distance between Lyn and bPAC(wt), by inserting an endoplasmic reticulum exit sequence and Venus, strongly reduced the dark cAMP. If a smaller Myc tag was introduced instead of Venus, dark activity was again apparent (Fig. 4A). Furthermore, we found that an extra repeat of Lyn in the N terminus increases membrane protein expression (Additional file 1: Figure S4, Fig. 4B) without influencing the enzymatic activity (Table 1). In vitro assay with extracted membrane also confirmed the reduced dark activity of Lyn-Venus-bPAC(wt) (Table 1). By introducing the F198Y point mutation into 2xLyn-Venus-bPAC, we obtained a membrane targeted PAC with no detectable dark activity and an L/D ratio higher than 4000 (Table 1). Plasma membrane localization of 2xLyn-Venus-bPAC(F198Y) was apparent in Hela cells, and we re-named this construct PACmn (Fig. 4C, Additional file 1: Figure S1). In oocytes expressing PACmn, no obvious cAMP accumulation (1.3 ± 0.2 μM) in the dark was observed. While 30 s light illumination increased cAMP to 31.7 ± 8.8 μM (Fig. 4A).

We tested additional N-terminal fusions to Venus-bPAC(wt) including the first 20 aa of GAP43, N-terminally modified QuasAr2 variants (lucy-Rho peptide or the “β helix” from the rat gastric H^+^-K^+^ ATPase), the G8R mutation of the Lyn peptide (MGCIKSKRKDS), and at the C-terminus CAAX (bPAC(F198Y)-T-mKate2-CAAX) [46–50]. These showed no improvement over PACmn in membrane localization or greatly reduced photocurrents and were not pursued further.

**Fig. 5:**
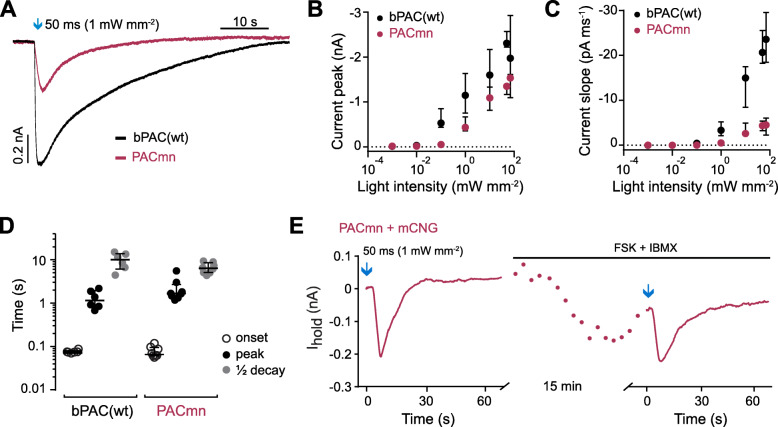
PACmn raises cAMP in hippocampal neurons. **A** Sample currents elicited by a 50-ms 470 nm light pulse (arrow) in neurons expressing either bPAC(wt) or PACmn together with mCNG. **B** Photocurrent amplitude and **C** slope recorded in neurons expressing PACmn or bPAC(wt) together with mCNG in response to 50 ms 470-nm light pulses of varying intensity. **D** Time from stimulation to onset, peak, and time from the peak to decay to ½ peak of responses to 50 ms, 1 mW mm^-2^ 470 nm light pulses. **E** Currents recorded from a neuron expressing PACmn together with mCNG in response to 50 ms, 1 mW mm^-2^ 470-nm light pulses (arrows), before and 15 min after wash-in of forskolin (FSK; 100 μM) and IBMX (75 μM). Plotted are median and interquartile range

When expressed in hippocampal neurons, PACmn was localized at the plasma membrane, with little to no immunostaining in the cytosol (Fig. 4D, Additional file 1: Figure S1). The Lyn peptide used in PACmn is known to target Src family kinases to the plasma membrane, in particular to lipid raft regions, and is likely responsible for the patchy appearance [51]. PACmn expressing neurons had normal morphology and electrophysiological properties (Fig. 4D–G). Notably, there was also no change in input or membrane resistance in neurons expressing bPAC(wt) together with a cAMP-sensitive channel (mCNG) suggesting that PKA is a more sensitive measure of increased resting cAMP than are tonic currents through mCNG channels. A short flash (50 ms) of blue light generated cAMP and inward currents in hippocampal neurons co-expressing mCNG with PACmn. Photocurrents were fully reversible and repeatable (Fig. 4H). PACmn photocurrents were light intensity dependent, with an EC50 of 4.1 mW mm^-2^ (Fig. 4I). Expectedly, lengthening the light pulse generated larger and longer lasting cAMP-evoked currents (Fig. 4J). In response to 50 ms light pulses, photocurrents in PACmn neurons were smaller than in bPAC(wt) neurons in response to 50 ms light flashes of the same light intensity but equal when intensity was increased 10x (Fig. 5A–C). Indeed when amplitude was saturated, the maximum photocurrents achieved with bPAC(wt) (−1.21 nA ± 0.21 nA, 50 ms 1 mW mm^-2^, Fig. 5A, B) were not different from maximum PACmn-induced photocurrents (−0.87 nA ± 0.20 nA, 500 ms 1mW mm^-2^, unpaired *t* test, from Fig. 4J). Thus, despite the reduced light-induced enzyme activity of PACmn, similar increases in cAMP are achieved by increasing either light intensity or duration. Photocurrents peaked within 1.7 s (PACmn) and 1.2 s (bPAC(wt)) from the start of illumination and decayed with a half-time of 6.4 s and 10 s, respectively (Fig. 5D). Similar to bPAC(wt) [13], currents generated by PACmn are larger than those produced by endogenous adenylyl cyclases activated with a saturating dose of forskolin (100 μM) and IBMX (75 μM) (Fig. 5E).

**Fig. 6:**
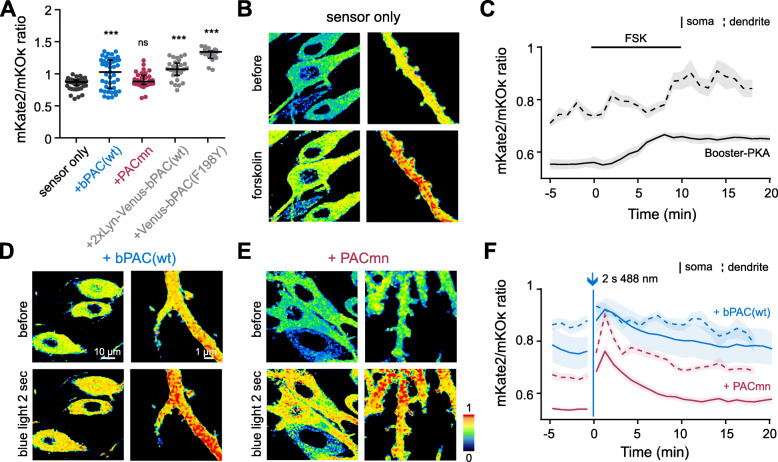
PACmn activates PKA when illuminated without altering basal activity in hippocampal neurons. **A** Resting FRET ratios (soma) in hippocampal neurons expressing Booster-PKA alone or together with bPAC variants. Note that only PACmn does not change resting PKA activity. Shown are median and interquartile range. ***p < 0.0001, ns = not significant, Dunnett’s multiple comparisons vs sensor only following one-way ANOVA (*p* < 0.0001). *n* = 52, 43, 31, 29, and 18 somata. **B** Representative ratio images (mKate2/mKoκ) of the hippocampal neurons (soma and dendrite with spines) expressing BoosterPKA before and after foskolin (FSK). **C** PKA activity in Booster-PKA expressing hippocampal neurons. Bar indicates time of FSK application. **D**, **E** Representative ratio images (mKate2/mKoκ) of hippocampal neurons expressing Booster-PKA together with bPAC(wt) (**D**) or PACmn (**E**) before and after being illuminated for 2 s with 1 mW mm^-2^ 470-nm blue light. **F** PKA activity in Booster-PKA neurons co-expressing bPAC(wt) or PACmn. Arrow indicates a 2 s blue light pulse. In both **C** and **F**, the solid lines are from the soma, dashed lines from the dendrites and spines. *n* (somata): 10 (Booster-PKA), 7 (+bPAC(wt)), 11 (+PACmn); *n* (dendrites and spines): 10 (Booster-PKA), 18 (+bPAC(wt)), and 14 (+PACmn). Shading indicates SEM. Results presented in **A** are from experiments done using a different microscope; thus, the difference in resting ratios in comparison to **C** and **F**

We next performed a side-by-side comparison of the resting (dark) PKA activity in neurons using Booster-PKA (Fig. 6A). Whereas co-expression of PACmn did not affect PKA activity, bPAC(wt) again raised resting PKA activity as did the 2xLyn-Venus-bPAC(wt) construct that lacks the F198Y mutation (Fig. 6A). Surprisingly, Venus-bPAC(F198Y) also increases PKA activity in neurons despite having no effect in the oocytes (Fig. 2B) or on *Drosophila* behavior (Fig. 3). In comparison to the slow, long-lasting effects of forskolin (Fig. 6B, C), a short (2 s) light activation of PACmn rapidly increased PKA activity which returned to baseline after about 10 min (Fig. 6E, F). We also confirmed that no cGMP was produced by PACmn (Additional file 1: Table S1). These results demonstrate the much lower dark activity of PACmn compared to bPAC(wt) and that PACmn can be used to activate cAMP-dependent intracellular signaling cascades in individual cells with high temporal and spatial resolution.

## Discussion

Modulation of intracellular cAMP concentration represents an essential step in multiple intracellular signaling pathways. Genetically encoded, photo-activatable cyclases (PACs) allow for a precise manipulation of cAMP in terms of spatial and temporal resolution in comparison to conventional pharmacology [11, 13]. Despite its widespread use, the enzymatic activity of bPAC(wt) has not yet been consistently measured. Turnover in the light has been reported as 0.05 s^-1^ for MBP-bPAC [14], 1.8 s^-1^ for SUMO-bPAC [38], and 2.6 s^-1^ for non-tagged bPAC [37], an increase of 100–300 fold over the cyclase activity in the dark. Instead of purifying the protein, we used soluble protein extracts from the oocytes that can be prepared in a much shorter time in gentler conditions. The light activity of bPAC we determined is 93 min^-1^ (1.5 s^-1^), a 1600-fold increase over the dark activity, which is in line with the values reported by Stierl et al. and Lindner et al. [37, 38]. Our determination of mPAC activity also agrees with the previous report [31]. We suggest that this simplified method of preparation is useful for determining enzymatic activity, albeit requiring the attachment of a fluorescent tag for protein quantification and a PDE inhibitor to accurately determine the dark activity. Other photochemical properties, such as light sensitivity and kinetics, are also reliably characterized from the extracts.

Albeit low, the dark activity of bPAC(wt) 0.057 min^-1^ (0.0009 s^-1^) is sufficient to increase resting cAMP in the absence of light, in agreement with other reports [44, 52, 53]. In consequence, about 25% of *Drosophila* larvae display nocifensive-like bending behavior in the absence of light when bPAC is expressed in nociceptors and bPAC-expressing hippocampal neurons have elevated resting PKA activity. Thus, bPAC sufficiently raises cAMP in the dark to activate downstream processes. Several alterations contributed to lowering the dark activity of bPAC. Attaching fluorescent proteins to either the N or C terminus, increasing the size of residue 198 from phenylalanine to tyrosine and anchoring to the membrane all decreased dark activity without abolishing light activity. Indeed the reduction in dark activity achieved with membrane anchoring and attachment of the fluorophores sufficed to prevent *Drosophila* expressing PACs from exhibiting nocifensive behavior in the dark. Similar to other adenylyl cyclases [54, 55], mutating the substrate binding residue (K197) or transition state stabilization residue (R278) significantly reduced, but did not abolish the catalytic activity of bPAC in both dark and light conditions. The high light to dark ratio of bPAC means detectable light activity remains with these mutations. Several other point mutations also decreased dark activity but also strongly decreased or abolished cyclase activity in the light (Fig. 2, Table 1). The reduction of dark activity reported for the S27A mutation was confirmed but we focused on mutations away from the flavin-binding domain to avoid changing the absorbance spectrum [38]. When soluble bPAC(R278A)-eYFP or Venus-bPAC(F198Y) was expressed in *Drosophila* nociceptive neurons, the larvae showed no nocifensive behavior in the dark and normal reactions to von Frey filament stimulation. Importantly, about 30% of the larvae exhibited the expected nocifensive responses when illuminated. This behavior occurred without noxious mechanical stimulation as a result of cAMP-dependent nociceptor sensitization. Curiously, with soluble Venus-bPAC(F198Y) both *Drosophila* and hippocampal neurons developed large vacuole-like bodies. In hippocampal neurons PKA activity, indicative of raised cAMP was observed when Venus-bPAC(F198Y) was expressed, despite that in PACmn the F198Y mutation is clearly necessary to lower resting PKA to undetectable levels and that Venus-bPAC(F198Y) did not alter oocyte cAMP. We currently have no explanation for why Venus-bPAC(F198Y) performs differently in oocytes and neurons. Possibly Venus-bPAC(F198Y) aggregates are formed by interaction with neuronal proteins not found in oocytes and this aggregation activates the cyclase. As bPAC(R278A)-eYFP showed normal cytoplasmic distribution and no dark nocifensive behavior in Drosophila neurons, we recommend this as a soluble PAC.

Tsvetanova et al. targeted bPAC(wt) (Lyn-bPAC aka bPAC-PM) to the plasma membrane, endosomes (bPAC-Endo), or cytoplasm (bPAC-Cyto) and found that differences in signaling depend on the subcellular location of cAMP generation [45, 56]. Our interpretation of the observed increase in dark activity of Lyn-bPAC is that when bPAC is directly fused to the membrane anchor, the orientation between the two bPAC molecules is altered changing the enzymatic activity. NanoLuc-bPAC, which is particularly interesting for in vivo applications where chemiluminescent bPAC activation is less invasive than direct light delivery, also has dark activity [57]. We suspect that adjusting/increasing the linkers and introducing either the F198Y, R278A (or other) mutations as we did for Lyn-bPAC may reduce the dark activity reported for not only NanoLuc-bPAC but also when bPAC is attached to other proteins such as cyclic nucleotide dependent channels [39, 58]. In our successful constructs, a fluorescent protein, eYFP, or Venus was the intervening “linker” and when Venus was replaced with a small HA tag in the Glycophorin-fusion construct, or with a myc tag in the Lyn construct, the dark activity increased supporting the notion that a large linker is necessary (Figs. 2, 4). In *Drosophila*, both the CD8-Venus-bPAC(wt) and Glycophorin-Venus-bPAC(S27A) caused nocifensive behavior only when the larvae were illuminated and therefore are suitable for studying cAMP-dependent processes.

In rat hippocampal neurons, better membrane localization was achieved when tandem Lyn11 sequences were used to target bPAC to the membrane. Unlike wild-type bPAC and the other variants tested, PACmn does not alter basal neuronal PKA activity but rapidly increases cAMP and PKA activity with blue light, demonstrating the superiority of PACmn over bPAC(wt) for investigating cAMP-dependent processes. Brief light stimulation of PACmn in neurons increased currents through cAMP-dependent CNG channels that were larger than those induced by forskolin and IBMX. The light-induced currents were not occluded by forskolin and IBMX but peaked within 2 s and returned to baseline within 1 min. PKA activation was also rapid but somewhat delayed, peaking after about 2 min and returning to baseline after 10 min. In contrast, forskolin stimulation of ACs required 10 min to peak and was not reversing 10 min later. Thus, light-activation of PACmn increases intracellular cAMP with cellular specificity, reversibility, and excellent temporal resolution.

## Conclusions

Our aim was to engineer a photoactivatable adenylyl cyclase that is targeted to the plasma membrane and that does not raise the resting cAMP concentration. Among the adenylyl cyclases tested, bPAC had the best properties. We found that adding a fluorescent tag and introducing certain point mutations further reduced dark activity in *Xenopus* oocytes. When expressed in *Drosophila* mechanosensory neurons, the F198Y and R278A variants induced nocifensive behavior under blue light but not in the dark or red light. In rat hippocampal neurons and *Xenopus* oocytes, we characterized PACmn, a membrane-targeted variant that no longer altered cAMP concentrations or PKA activity in the dark but dramatically and rapidly reversibly increased intracellular cAMP and activated PKA with brief flashes of blue light. We conclude that PACmn is a versatile optogenetic tool for precisely and specifically increasing cAMP in various cell types.

## Methods

All experiments involving animals were performed according to German law (Tierschutzgesetz der Bundesrepublik Deutschland, TierSchG) and approved by the respective authorities and ethics committees. In Hamburg from the Behörde für Justiz und Verbraucherschutz (BJV)-Lebensmittelsicherheit und Veterinärwesen, Hamburg and the animal care committee of the UKE. In Würzburg, *Xenopus* laevis surgery for oocytes was under permission of Regierung of Unterfranken. Statistical analyses were performed using GraphPad Prism and detailed results are presented in **“**Additional file 2: Spreadsheets of numerical data of Figures 1, 2, 3, 4, 5, 6 and Figure S3”.

### Molecular biology and plasmids

#### *Xenopus* oocytes

The DNA of bPAC-eYFP and Venus-bPAC were cloned into the oocyte expression vector pGEM-HE by USER cloning. All bPAC point mutations were introduced by quick change PCR. The rest tested PAC variants were inserted into pGEMHE within N-terminal BamHI and C-terminal HindIII restriction sites. All the constructs were confirmed by sequencing. After plasmid linearization by NheI digestion, cRNAs were generated with the AmpliCap-MaxT7 High Yield Message Maker Kit (Epicentre Biotechnologies). The following plasmids for cRNA production are available from Addgene: pGEM-HE-Venus-bPAC(F198Y) Addgene #165487, pGEM-HE-bPAC(R278A)-Myc-eYFP Addgene #165488, pGEM-HE-2xLyn-ERex-Venus-bPAC(F198Y)/PACmn Addgene #165489, and pGEM-HE-Glyco-Venus-bPAC(S27A) Addgene #165490. Other plasmids are available by request from S. Gao.

#### Drosophila

The following strains were used:

- *w*^*1118*^ (control)
- w*; ppk-GAL4 (BDSC#32078)
- w*; ok6-GAL4 [59]
- *w*^*1118*^*; UAS-bPAC/CyOGFP w*^*-*^ [13]

The following genotypes were generated for this study:

- *y*^*1*^ *w*^*1118*^*; 20XUAS-bPAC(R278A)::eYFP/CyO* (RJK559)
- *y*^*1*^ *w*^*1118*^*; 20XUAS-CD8::Venus::bPAC/CyO* (RJK560)
- *y*^*1*^ *w*^*1118*^*; 20XUAS-Glyco::Venus::bPAC(S27A)/CyO* (RJK563)
- *y*^*1*^ *w*^*1118*^*;+; 20XUAS-Venus::bPAC(F198Y)/Sb* (RJK1007)

#### Rat hippocampal neurons

For use in hippocampal slice cultures, DNA inserts were cloned from the pGEM plasmids used in oocyte recordings into pAAV-Synapsin expression vectors via EcoRI and Acc65I (primers: 5′-TAGTGGTAACCAGATC-3′, 5′-CACTGGAGCTATCAACGGAG-3′). The following is available on Addgene: pAAV-syn-2xLyn-ERex-Venus-bPAC(F198Y)/ PACmn (#205) Addgene #165491. Additional plasmids used were pCI-syn-mKate2 re-cloned from Evrogen FP182, pCI-syn-CNGA2(C460W/E583M) recloned from gift of J Karpen [13], pCI-syn-bPAC-myc (#DU5) re-cloned from Addgene #28134 [13], pCAGGS-4493NES (Booster-PKA) Addgene #13837, pAAV-syn-Glyco-Venus-bPAC(S27A)-myc, pAAV-syn-2xLyn-ERex-Venus-bPAC(wt), and pAAV-syn-Venus- bPAC(F198Y)-myc.

### Oocyte experiments

#### Oocyte injection

Stage V or VI *Xenopus* oocytes were prepared in Julius-von-Sachs Institute, Würzburg. After the injection of cRNAs, oocytes were maintained in the dark for 3 days at 18°C in ND96 solution: 96 mM NaCl, 2mM KCl, 1 mM CaCl_2_, 1 mM MgCl_2_, 10 mM HEPES, and 50 μg/ml gentamycin pH 7.4. The amount of cRNA injected per oocyte in Figs. 1A; 2A, D, and E; and 4A–B was adjusted to keep copy number approximately equal: bPAC, Lyn-bPAC, and 2xLyn-Myc-bPAC (20 ng); Glyco-HA-bPAC (25 ng); Venus or eYFP tagged soluble bPAC variants, Lyn-Venus-bPAC, and 2xLyn-Venus-bPAC (30 ng); and CD8-Venus-bPAC and Glyco-Venus-bPAC (35 ng). For the remaining PACs, the cRNA injection quantity is 30 ng/oocyte unless otherwise indicated.

#### cAMP ELISA assay from whole oocyte lysates

Oocytes were either kept in the dark or illuminated for 30 s with 473 nm blue light at 0.3 mW mm^-2^ before homogenization. Under far-red light, five oocytes were pooled and homogenized by pipetting in 0.1 N HCl. Cell debris was removed by 10 min 30,000*g* centrifugation at room temperature. The supernatant was collected and cAMP was quantified using the DetectX High Sensitivity Direct Cyclic AMP Chemiluminescent Immunoassay Kit (Arbor Assays).

#### In vitro cyclase activity assay with soluble proteins

Fifteen control oocytes (without cRNA injection) or 15 oocytes pre-injected with cRNA encoding soluble bPAC variants were pooled and washed 3 times in ice-cold solution A (75 mM Tris-HCl, 100 mM NaCl, 2 mM MgCl_2_, 5 mM DTT, 1x Protease Inhibitor Cocktail (Roche), pH 7.4). After gentle homogenization by pipetting in 450 μl ice-cold solution A, the yolk and cellular debris were sedimented by 30,000×*g* centrifugation at 4°C for 20 min. The lipid layer on the surface of the supernatant was removed, and the supernatant transferred to a new tube and centrifuged at 30,000×*g* at 4°C for another 20 min to remove the residual lipid layer. The oocyte soluble extract (supernatant) was then ultrafiltrated by centrifugation with the Amicon Ultra-0.5 Centrifugal Filter Unit. Small proteins, endogenous ATP/GTP, cAMP/cGMP, and other small molecules were removed by the ultrafiltration step. The crude soluble extracts were then ready for the in vitro reactions. This preparation process was performed under red light.

To determine the endogenous PDE activity, 0.15 μM cAMP was added to the crude soluble extracts with or without IBMX. To determine either endogenous or soluble PAC-induced adenylyl cyclase activity, reactions were started by adding ATP (final concentration 1 mM) to the crude soluble extracts in the dark (under far-red light) or blue light as indicated in the figure legends.

Aliquots were taken from the in vitro reaction mix and stopped by adding 19 volumes of Sample Diluent (containing 0.1 N HCl, Arbor Assays) at the indicated time points (in Fig. 1B and C). For reaction with the soluble PAC expressing crude extracts, the aliquots were taken and stopped at 3 different time points in the light (1, 4, and 7 min) and in the dark (5, 25, and 45 min). cAMP was quantified using the DetectX High Sensitivity Direct Cyclic AMP Chemiluminescent Immunoassay Kit (Arbor Assays) and turnover calculated after protein quantification (as below).

#### Membrane cyclase in vitro activity assay

Fifteen oocytes (uninjected controls or expressing membrane-targeted PACs) were pooled and washed 3 times in ice-cold solution A (see above). After gently homogenizing in 450 μl solution A by pipetting on ice, the yolk and cellular debris were sedimented by 500×*g* centrifugation at 4°C for 15 min. The supernatant was transferred to a new tube and centrifuged at 30,000×*g* at 4°C for 20 min for separation of membrane pellets and soluble fraction. The supernatant was discarded. The membrane pellets were gently washed twice with 500 μl solution A and resuspended in solution A at a ratio of 1 oocyte to 4 μl. The resuspended fraction was again centrifuged 500×*g* at 4°C for 5 min to remove any residual large cellular debris. The supernatant (membrane extract suspension) was used for the in vitro reaction. To start the enzymatic reaction, 4 μl membrane extract was mixed with 36 μl reaction buffer (75 mM Tris-HCl, pH 7.4, 100 mM NaCl, 5 mM DTT, 5 mM MgCl_2_, 1 mM ATP, 0.2 mM GTP) either in black Eppi tubes (the dark condition) or under illumination (473 nm, 0.3 mW mm^-2^) from the top of the Eppi tube. Aliquots were taken from the in vitro reaction mix and stopped as the soluble protein assay. cAMP concentration was quantified using the DetectX High Sensitivity Direct Cyclic AMP Chemiluminescent Immunoassay Kit (Arbor Assays). The enzyme turnover (μM cAMP/μM enzyme/minutes from start of reaction) was calculated after the fluorescence-based protein quantification (below).

#### Fluorescence-based protein quantification

The fluorescence emission of purified Venus and eYFP protein (Evrogen JSC; 1 mg/ml) of known concentration (dilution to 0, 5, 10, 20, 40, 80, and 160 ng with solution A, three repeats) was measured at 538 nm by using a Fluoroskan Ascent microplate fluorometer with 485 nm excitation and used to plot standard curves. The protein levels in *Xenopus* oocyte membranes and soluble extracts were obtained by measuring fluorescence of the samples on the same fluorometer and comparing to the standard curves.

#### Oocyte imaging

Venus fluorescence imaging of oocytes was done using a confocal laser scanning microscope (LSM 5 Pascal, Carl Zeiss) equipped with a Zeiss Plan-Neofluar 10× 0.5 nA objective. Images were processed using LSM 5 Image Browser and exported for insertion into figures.

### Expression and imaging in Hela229 cells

HeLa229 cells (ATCC CCL-2.1^TM^) were grown at 37°C in a humidified atmosphere containing 5% (v/v) CO_2_ in 10% (v/v) heat inactivated fetal bovine serum (FBS, Sigma-Aldrich) RPMI1640 + GlutaMAX^TM^ medium (Gibco^TM^) complemented with sodium pyruvate (Gibco^TM^). For microscopy, the cells were seeded on 15 mm coverslips (VWR) in 12-well plates (Corning) 1 day prior transfection with plasmids encoding CD8-Venus-bPAC(wt), Glyco-Venus-bPAC(wt), and 2xLyn-Venus-bPAC(F198Y). Transfection was performed using Viromer® RED (230155; Biozym, Oldendorf, Germany) according to manufacturer’s instructions. Twenty-four h after adding the transfection mix, the cells were fixed using 4% PFA in PBS (Morphisto), washed 3 times with 1xPBS, and then mounted onto glass-slides using 2.5% Mowiol-DABCO (Carl Roth, Karlsruhe, Germany). Imaging was performed on a Zeiss (Oberkochen, Germany) ELYRA S.1 SR-SIM structured illumination platform using a Plan-Apochromat 63x/1.4 Oil DIC M27 objective with a numerical aperture of 1.4. Reconstruction of super-resolution images was performed using the ZEN image-processing platform with a SIM module. The images were processed using Fiji 1.51n [60].

### *Drosophila* experiments

Flies were raised on standard cornmeal and molasses medium at 25°C.

#### Immunohistochemistry

Expression studies in *Drosophila* larvae were performed as previously described [61]. Third-instar larvae of appropriate genotypes were dissected in ice-cold hemolymph-like HL-3 saline [62] followed by fixation with 4% PFA for 10 min. The fillets were washed in 1x PBS and blocked with 5% NGS in 0.05% PBST for 30 min at room temperature. Primary staining was done with either mouse anti-GFP antibody (1:500; Sigma-Aldrich, SAB4200681; RRID:AB_2827519) or mouse anti-Brp (nc82, 1:100; RRID: AB_528108) with 5% NGS in 0.05% PBST at 4°C, overnight. The following day, the fillets were washed with 0.05% PBST (2 fast washes and 3 washes, 20 min each) before being treated with the secondary antibody. Secondary antibodies Alexa Fluor-488-conjugated goat-α-mouse (1:250; Invitrogen, A-11001; RRID:AB_2534069) and Cy3 conjugated anti-Horseradish Peroxidase (HRP; 1:250; Jackson ImmunoResearch Labs 123-165-021, RRID:AB_2338959) were added and incubated for 2 h at room temperature. After washing again, the fillets were stored in Vectashield (Vector Laboratories) overnight at 4°C. Subsequently, the fillets were mounted and imaged using a Zeiss LSM 800 confocal microscope fitted with a Plan-Neofluar 16x/0.50 oil immersion and Plan-Apochromat 63x/1.4 oil immersion objective and the appropriate lasers. The images were processed in Fiji [60], and analysis of NMJ parameters was performed as described before [61].

#### Nocifensive response

The nocifensive behavioral response was assessed as described in Dannhäuser et al. 2020 [44]. Wandering third-instar larvae were placed in a drop of water on a Sylgard-coated Petri dish and observed through a stereomicroscope (Leica M80). For dark conditions, the experiment was performed under red light. bPAC activation was achieved with blue light (470 nm) of ~500 μW mm^-2^ for 3 min. Larvae exhibiting head swinging or bending without noxious mechanical stimulation were graded as bending and removed. Larvae exhibiting nocifensive-like rolling were graded as rolling and removed. To the remaining larvae, a rapid single noxious stimulus was delivered with a 40 mN von Frey filament (made with Caperlan fishing line of Ø 0.22mm, 4.11 kg tensile strength) to the dorsal side (abdominal segments A4-A6). Larvae that reacted with at least one stereotypical corkscrew roll along the longitudinal axis were graded as positive responses to the von Frey stimulation. Each animal was scored only once and approximately 50–200 larvae were tested per genotype.

### Rat hippocampal neuron experiments

#### Hippocampal slice cultures and transfection

Hippocampal slice cultures were prepared from P5–P7 female Wistar rats (Unilever Wistar HsdCpd:Wu, Envigo) and maintained without antibiotics as described [63]. After at least 7 days in vitro, CA3 neurons were transfected by single-cell electroporation [64]. Neurons were co-electroporated with DNA encoding one of the cyclases (10 ng/μl) together with DNA encoding the far-red fluorescent protein mKate2 (20 ng/μl) and, where specified, the cAMP-sensitive CNG-A2 channel (C460W/E583M) (*K*_1/2_^cAMP^ = 0.89 μM, *K*_1/2_^cGMP^ = 6.2 μM) [65]. Alternatively, for FRET imaging experiments, CA3 neurons were electroporated with DNA encoding the PKA FRET sensor Booster-PKA (25 ng/μl) alone or with DNA encoding a cyclase (25 ng/μl).

#### Rat hippocampal neuron electrophysiology

Whole-cell patch-clamp recordings (holding voltage −70 mV) were performed 7–10 days after transfection using an Axopatch 200B amplifier (Molecular Devices). National Instruments A/D boards and Ephus software running in Matlab were used to record and control the experiment [66]. The microscope (Olympus BX61WI) was fitted with an LED (Mightex Systems), which was coupled through the camera port using a multimode fiber (1.0 mm) and collimator (Thorlabs) to photo-stimulate through the 40 x water immersion objective (Plan-Apochromat, 40x 1.0 numerical aperture, Zeiss). Radiant power was determined using a power meter and silicon detector (Newport 1936R, 818-ST2) positioned in the specimen plane and divided by the illuminated field (0.244 mm^2^).

The extracellular solution contained (in mM): NaCl 119, NaHCO_3_ 26.2, d-glucose 11, KCl 2.5, NaH_2_PO_4_ 1, MgCl_2_ 4, CaCl_2_ 4, pH 7.4, 310 mOsm kg^-1^, and saturated with 95% O_2_/5% CO_2_. Recording temperature was 29 ± 1°C, and a mix of picrotoxin (100 μM), NBQX (10 μM), and CPPene (10 μM) (Tocris) was added to the extracellular solution to block synaptic responses. Where indicated, forskolin (FSK; 100 μM) and 3-isobutyl-1-methylxanthine (IBMX; 75 μM) (HelloBio) were added from concentrated stock solutions or an equivalent amount of dimethylsulfoxide (DMSO). The intracellular solution contained (in mM): K-gluconate 135, HEPES 10, EGTA 0.2, Na_2_-ATP 4, Na-GTP 0.4, MgCl_2_ 4, ascorbate 3, Na_2_-phosphocreatine 10, pH 7.2, and 295 mOsm kg^-1^. The liquid junction potential was measured (−14.4 mV) and compensated. Patch electrodes were made from thick-walled borosilicate glass and had resistances of 3–5 MΩ when filled. Series resistance during the recordings was less than 15 MΩ and was not compensated during voltage-clamp recordings. The bridge balance compensation circuitry was used during current-clamp recordings. Analysis of the photocurrents was performed using custom Matlab scripts, while graphs and statistical analyses were generated with GraphPad Prism 6.0. Bars and whiskers on graphs represent median and interquartile range of the data if not otherwise stated. The slope of the currents was calculated from 30 to 50% of the response amplitude.

#### Immunohistochemistry and microscopy

Hippocampal slice cultures with neurons expressing PACmn (2xLyn-Venus-bPAC(F198Y)) and mKate2 were fixed in 4% PFA in PBS for 30 min then washed three times with PBS. After incubation for 2 h at room temperature in blocking buffer (0.3% TritonX, 5% goat serum in PBS), slices were placed in primary antibody (Chicken Anti GFP polyclonal antibody 1:500, Invitrogen (A10262, Lot 1972783)) solution overnight at 4°C (0.3% TritonX, 5% goat serum, 1% BSA in PBS). After three 10-min washes with PBS, slices were incubated for 2 h at room temperature with secondary antibody (Alexa Fluor 488 conjugated secondary antibody (goat anti-chicken, 1:500, Life technologies; A11039) solution (0.3% TritonX, 5% goat serum, 1% BSA in PBS). Prior to mounting, the slices were washed three times with PBS.

Images of stained slices were acquired with a confocal laser scanning microscope (Olympus FLUOVIEW FV1000) using 20x and 60x oil immersion objectives (UPLSAPO 20X NA: 0.85, 60X NA: 1.35). A stack of multiple images (0.5–3 μm step) was acquired using the 488 and 559 nm laser lines. In order to avoid spectral bleedthrough, acquisition was done sequentially.

#### PKA activity measurements in the hippocampal neurons using Förster resonance energy transfer (FRET) imaging

Five to 7 days after electroporation, neurons expressing the PKA sensor Booster-PKA alone or together with bPAC(wt), PACmn, 2xLyn11-Venus-bPAC(wt), or Venus-bPAC(F198Y) were imaged using confocal laser scanning microscopy.

Experiments in Figs. 1F and 6C, D were done at 32°C using an Olympus FLUOVIEW FV1000 microscope. A 559-nm laser line was used for excitation, and donor/acceptor channel filters were set at 560–600 nm and 640–740 nm. All acquisition parameters were the same throughout all imaging sessions. Multiple *Z* planes were acquired (step size 0.5 μm) every minute. After 5 min of baseline, the whole field of view was scanned for 2 s with the 488-nm laser line to activate the expressing cyclases. The 488-nm activation ended 1 s before the next 559-nm signal acquisition. Alternatively, forskolin (50 μM) was washed in to activate endogenous membrane bound adenylyl cyclases. One *Z* plane was chosen for the analysis. Image analysis was done using Fiji software [60]. After background removal, a translational alignment was applied using the StackReg plugin [67] in order to align the time series in X and Y dimensions. Appropriate regions of interest were selected (soma or dendrites with spines), and the mean intensity of donor and acceptor channels were measured.

Experiments in Fig. 6A were done at a different Olympus FLUOVIEW FV1000 microscope at room temperature. A 559-nm laser line was used for excitation, and donor/acceptor channels were set at 560–600 nm and 640–740 nm. Multiple *Z* planes were acquired (step size 0.5 μm), and a maximum projection was used for analysis (using Fiji). After background removal, regions of interest were selected (somata) and the mean intensity of donor and acceptor channels were measured.

For both sets of experiments, the ratios reported were calculated from the individual donor and acceptor channel measurements, while the ratio images were generated using the RatioPlus plugin of Fiji.

## Supplementary Information

**Additional file 1: Figure S1.** bPAC variants in HeLa229 cells and hippocampal neurons. **Figure S2.** Expression of bPAC variants in *Drosophila* motoneurons. **Figure S3.** Motoneuron morphology of *Drosophila* larvae. **Figure S4.** Expression of Lyn-Venus-bPAC(wt) and 2xLyn-Venus-bPAC(wt) in *Xenopus* oocytes. **Table 1.** cGMP is not produced by bPAC variants. **Table 2.** Statistical results.

**Additional file 2.** Spreadsheets of numerical data in Figures 1, 2, 3, 4, 5, 6 and Figure S3.

## Data Availability

The datasets supporting the conclusions of this article are included within the article and its additional files. The following plasmids used in this study are available on Addgene (other plasmids are available by request from S. Gao.): • pGEM-HE-Venus-bPAC(F198Y) Addgene #165487 • pGEM-HE-bPAC(R278A)-Myc-eYFP Addgene #165488 • pGEM-HE-2xLyn-ERex-Venus-bPAC(F198Y) /PACmn Addgene #165489 • pGEM-HE-Glyco-Venus-bPAC(S27A) Addgene #165490 • pAAV-syn-2xLyn-ERex-Venus-bPAC(F198Y) /PACmn Addgene #165491
