# Supplementary material for: PACmn for improved optogenetic control of intracellular cAMP
Source: BMC Biol. 2021 Oct 18;19:227. doi: 10.1186/s12915-021-01151-9 (PMC8522238; doi:10.1186/s12915-021-01151-9)
Supplement: Supplementary file 1 — Additional file 1: Figure S1. bPAC variants in HeLa229 cells and hippocampal neurons. Figure S2. Expression of bPAC variants in Drosophila motoneurons. Figure S3. Motoneuron morphology of Drosophila larvae. Figure S4. Expression of Lyn-Venus-bPAC(wt) and 2xLyn-Venus-bPAC(wt) in Xenopus oocytes. Table 1. cGMP is not produced by bPAC variants. Table 2. Statistical results. [file 12915_2021_1151_MOESM1_ESM.docx]

###### **
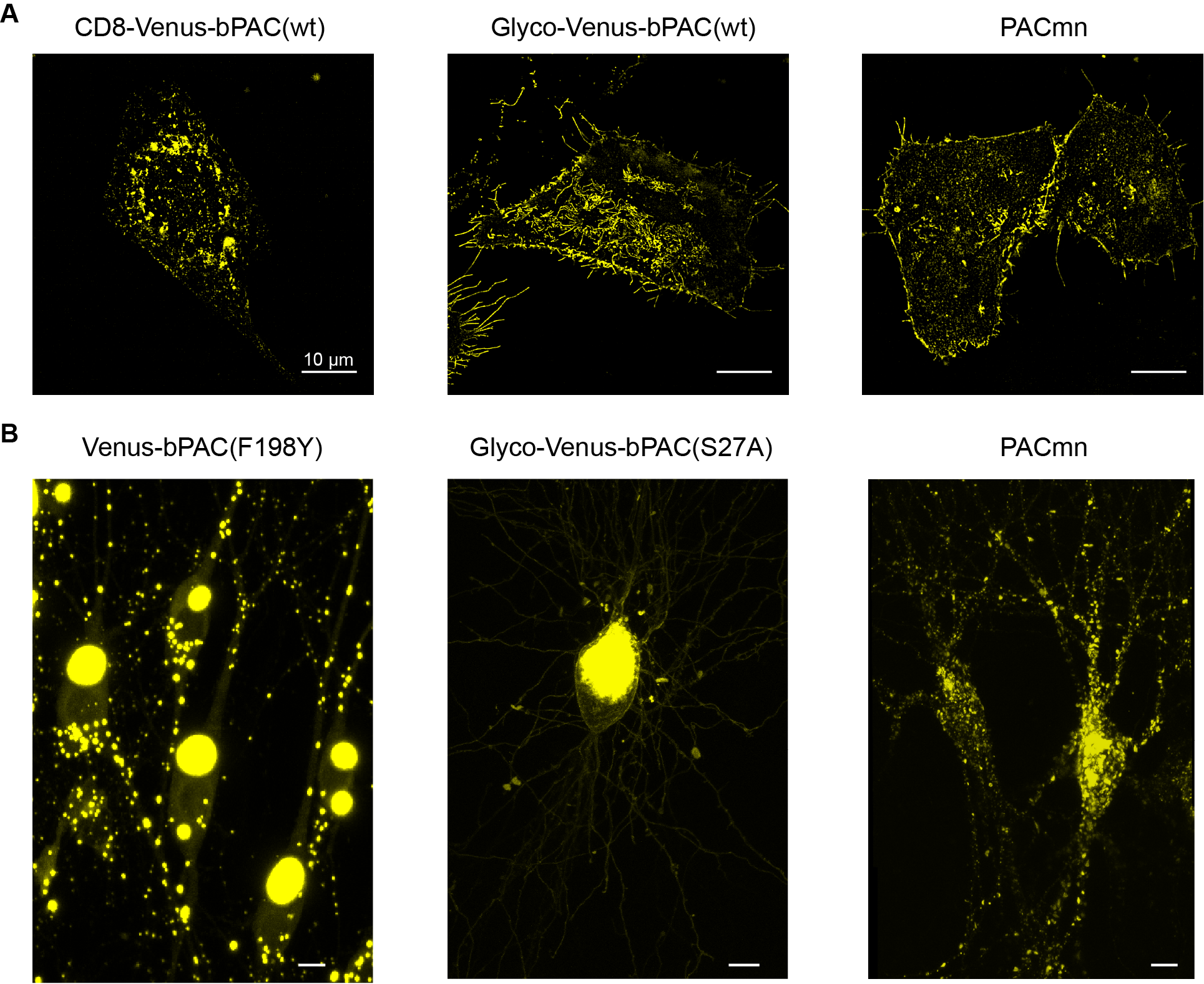
**

###### **Additional file 1: Fig. S1. bPAC variants in HeLa229 cells and hippocampal neurons.**

**A)** Human HeLa229 cells (ATCC CCL-2.1^TM^) were cultured in 10% (v/v) heat inactivated FBS (Sigma-Aldrich) RPMI1640 + GlutaMAX^TM^ medium (Gibco^TM^). The cells were grown in a humidified atmosphere containing 5% (v/v) CO_2_ at 37 °C. For microscopy, HeLa229 cells were grown on coverslips in 12-well plates and, upon adhesion, transfected with plasmids encoding CD8-Venus-bPAC(wt), Glyco-Venus-bPAC(wt) and PACmn (2xLyn-Venus-bPAC(F198Y)) using Viromer® RED (230155; Biozym, Oldendorf, Germany) according to manufacturer’s instructions. 24 h post transfection, the cells were fixed with 4% PFA and then mounted onto glass-slides using 2.5% Mowiol-DABCO (Carl Roth, Karlsruhe, Germany). Images were acquired on a Zeiss (Oberkochen, Germany) ELYRA S.1 SR-SIM structured illumination platform using a PLAN-Apochromat 63x oil-immersion objective with a numerical aperture of 1.4. Reconstruction of super-resolution images was performed using the ZEN image-processing platform with a SIM module. A single plane from the bottom of the cells is shown. Note the improved plasma membrane localization of PACmn. **B)** Live hippocampal neurons expressing Venus-tagged bPAC variants. Maximum intensity projections of confocal LSM images.


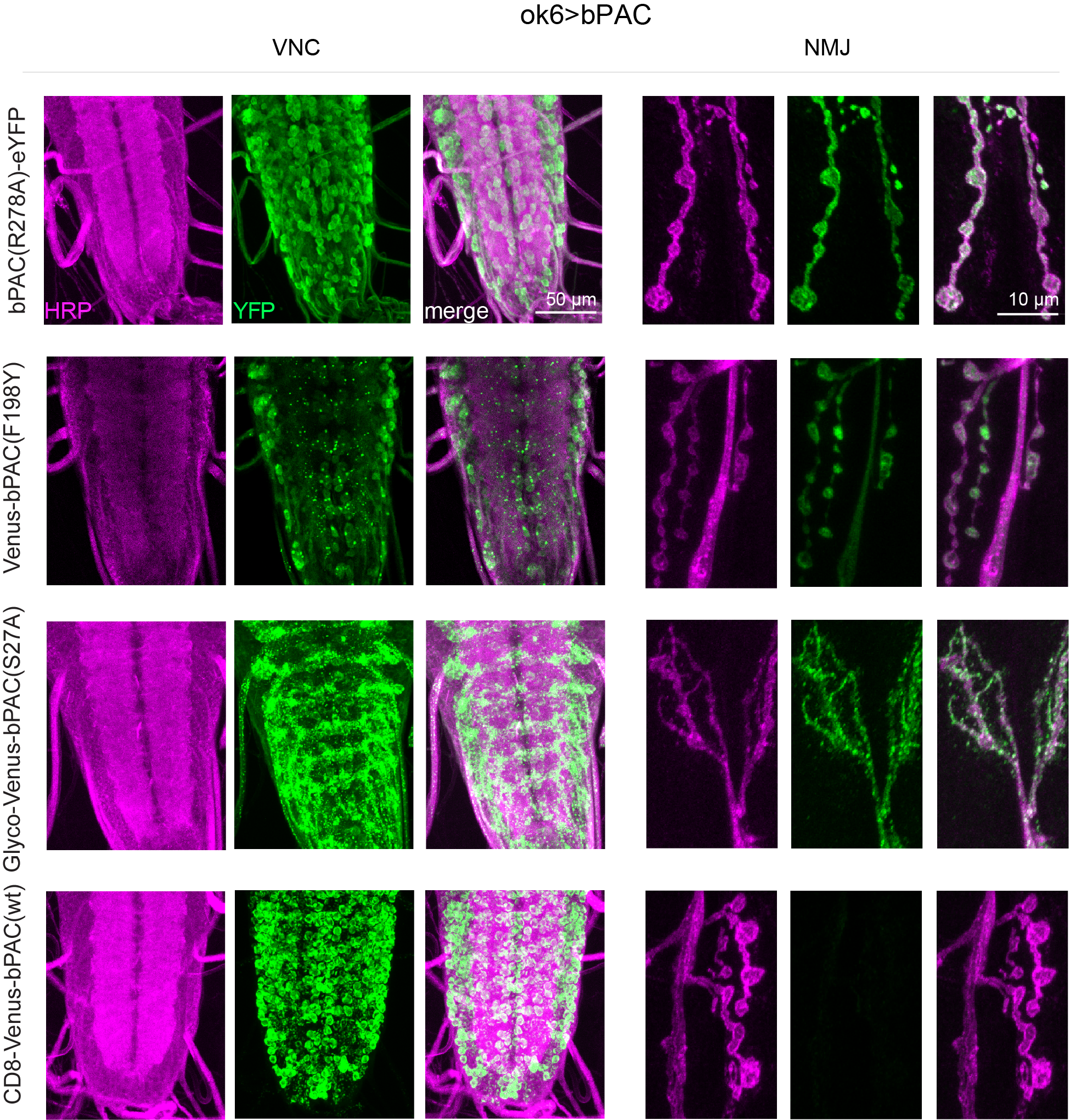


###### **﻿Additional file 1: Fig. S2. Expression of bPAC variants in Drosophila motoneurons**

Expression of bPAC variants (green) in *Drosophila* motoneurons (*ok6-GAL4* driver) labelled with HRP (horseradish peroxidase, magenta). Ventral nerve cord (VNC, arrowheads indicate axons), neuromuscular junction (NMJ). Note that the soluble and Glyco-Venus-bPAC(S27A) are expressed throughout the motoneurons, whereas CD8-Venus-bPAC(wt) is absent from the NMJ.

## **
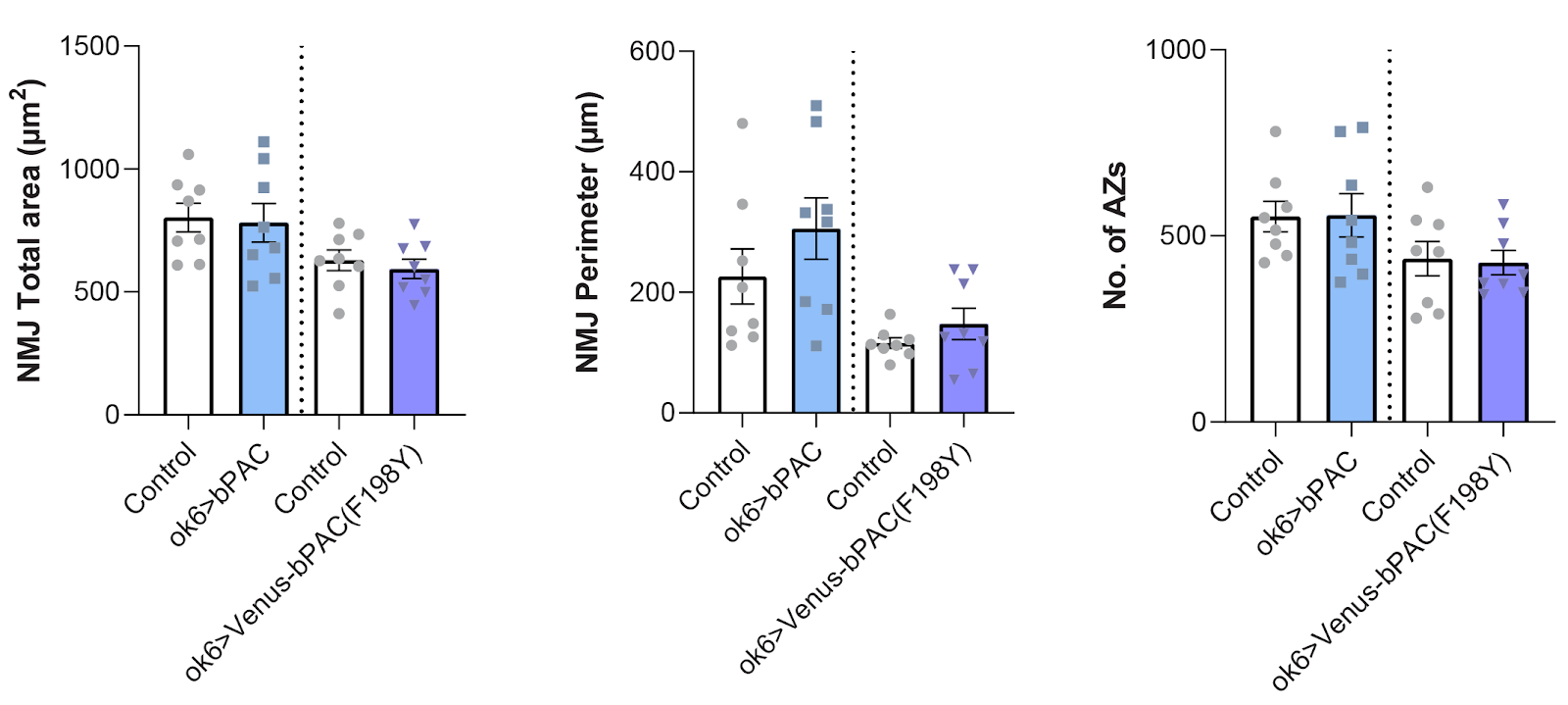
**

###### **Additional file 1: Fig. S3. Motoneuron morphology of Drosophila larvae.**

Imaging by confocal microscopy shows that neither the size of the NMJ (motoneurons labelled with the membrane marker anti-HRP), nor the number of presynaptic active zones (AZs, labelled with anti-Brp) are altered in bPAC expressing larvae [*ok6-GAL4>UAS-bPAC* and *ok6-GAL4>UAS-Venus-bPAC(F198Y)*] raised in the dark.

######
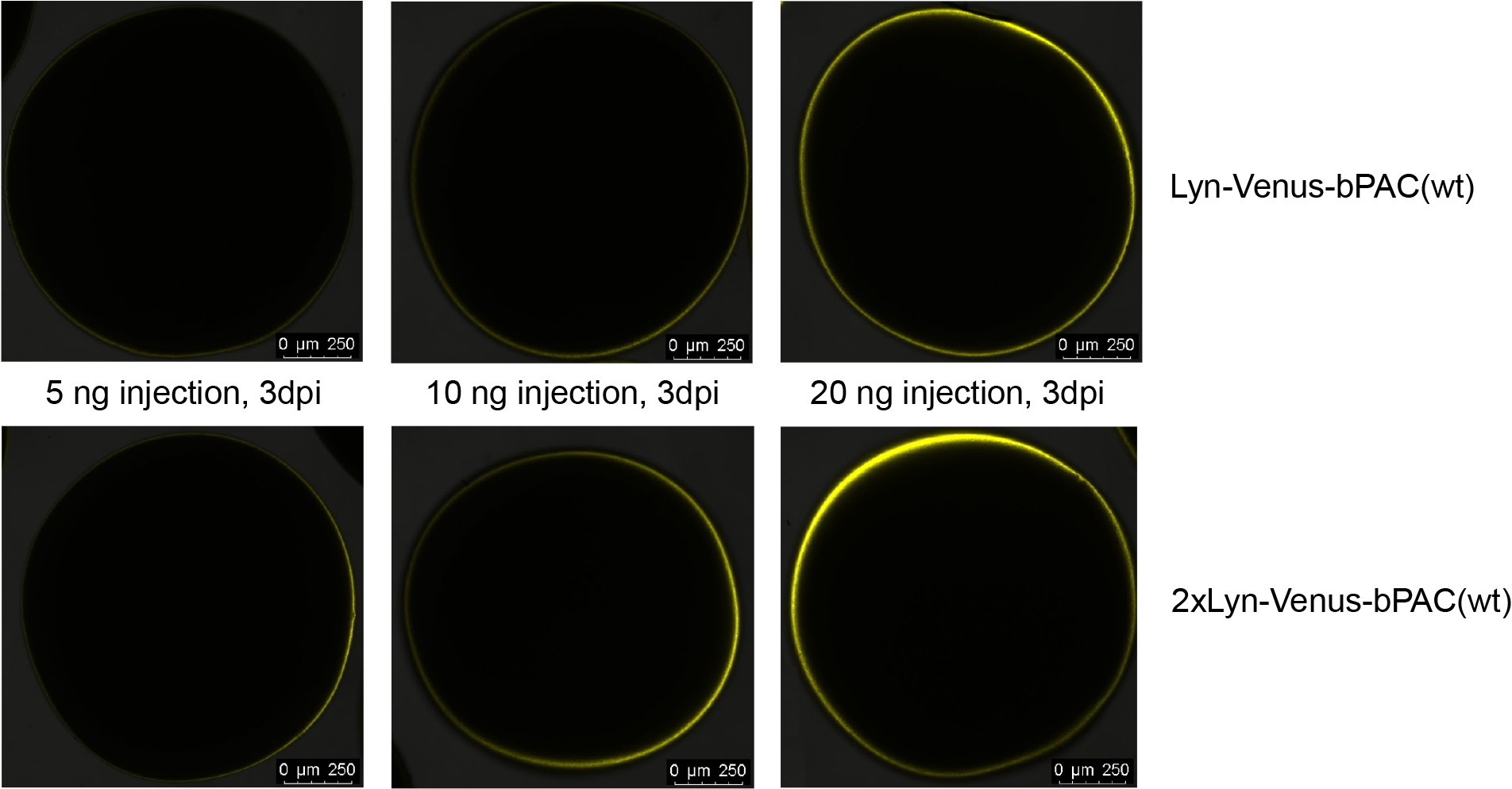
**Additional file 1: Fig. S4. Expression of Lyn-Venus-bPAC(wt) and 2xLyn-Venus-bPAC(wt) in Xenopus oocytes.**

Oocytes were injected with the indicated amount of cRNAs of Lyn-Venus-bPAC(wt) and 2xLyn-Venus-bPAC(wt). After 3 days of expression, oocytes were imaged by confocal microscopy. Shown are single planes through the center of the oocyte.

###### **Additional file 1: Table 1. cGMP is not produced by bPAC variants**

| **Condition**  (light = 1 min blue illumination) | **cGMP (µM)**  average (individual measurements, 8 oocytes per measurement) |
| --- | --- |
| control | 0.455 (0.51, 0.4) |
| Venus-bPAC(wt) (dark) | 0.48 (0.47, 0.49) |
| Venus-bPAC(wt) (light) | 0.475 (0.51, 0.44) |
| Glyco-Venus-bPAC(S27A) (dark) | 0.48 (0.49, 0.47) |
| Glyco-Venus-bPAC(S27A) (light) | 0.49 (0.44, 0.54) |
| *2xLyn-Venus-bPAC(F198Y) (dark) | 0.475 (0.56, 0.39) |
| *2xLyn-Venus-bPAC(F198Y) (light) | 0.445 (0.46, 0.43) |
| *PACmn | |

######

###### **Additional file 1: Table 2. Statistical results**

| **Figure** | | **Test** | **P value** | **Notes** |
| --- | --- | --- | --- | --- |
| **1** | **a** | ANOVA | < 0.0001 |  |
|  |  | Dunnett’s multiple comparison | < 0.0001 | bPAC dark vs. control dark |
|  |  |  | 0.021 | Venus-bPAC dark vs. control dark |
|  |  |  | 0.021 | bPAC-eYFP dark vs. control dark |
|  | **f** | Unpaired t-test | < 0.0001 | bPAC soma vs. sensor only soma |
|  |  | Unpaired t-test | 0.0006 | bPAC dendrite vs. sensor only dendrite |
| **2** | **b** | ANOVA | < 0.0001 |  |
|  |  | Dunnett’s multiple comparison | > 0.9999 | H2O vs. control dark |
|  |  |  | < 0.0001 | Venus-bPAC(wt) dark vs. control dark |
|  |  |  | < 0.0001 | bPAC(wt)-eYFP dark vs. control dark |
|  |  |  | 0.9860 | S27A dark vs. control dark |
|  |  |  | 0.9995 | L123R dark vs. control dark |
|  |  |  | 0.9999 | K197A dark vs. control dark |
|  |  |  | > 0.9999 | K197A/D201A dark vs. control dark |
|  |  |  | 0.9998 | F198Y dark vs. control dark |
|  |  |  | 0.9999 | F198W dark vs. control dark |
|  |  |  | 0.2937 | H266W dark vs. control dark |
|  |  |  | 0.9999 | T267Y dark vs. control dark |
|  |  |  | 0.9999 | R278A dark vs. control dark |
|  | **d** | ANOVA | < 0.0001 |  |
|  |  | Dunnett’s multiple comparison | < 0.0001 | Venus-bPAC(wt) dark vs. control dark |
|  |  |  | 0.9925 | CD8-Venus-bPAC(wt) dark vs. control dark |
|  |  |  | 0.9998 | Glyco-Venus-bPAC(wt) dark vs. control dark |
|  |  |  | < 0.0001 | Glyco-HA-bPAC(wt) dark vs. control dark |
|  | **e** | ANOVA | < 0.0001 |  |
|  |  | Dunnett’s multiple comparison of membrane fractions | 0.9831 | Control mb. vs. Venus-bPAC(wt) |
|  |  |  | < 0.0001 | CD8-Venus-bPAC(wt) vs. Venus-bPAC(wt) |
|  |  |  | < 0.0001 | Glyco-Venus-bPAC(wt) vs. Venus-bPAC(wt) |
| **4** | **a** | ANOVA | < 0.0001 |  |
|  |  | Dunnett’s multiple comparison | 0.0089 | Venus-bPAC(wt) dark vs. control dark |
|  |  |  | < 0.0001 | Lyn-bPAC(wt) dark vs. control dark |
|  |  |  | > 0.9999 | Lyn-Venus-bPAC(wt) dark vs. control dark |
|  |  |  | < 0.0001 | 2xLyn-myc-bPAC(wt) dark vs. control dark |
|  |  |  | 0.9999 | 2xLyn-Venus-bPAC(wt) dark vs. control dark |
|  |  |  | 0.9999 | PACmn dark vs. control dark |
|  | **b** | ANOVA | < 0.0001 |  |
|  |  | Dunnett’s multiple comparison of membrane fractions | 0.9989 | Control vs. Venus-bPAC(wt) |
|  |  |  | < 0.0001 | Lyn-Venus-bPAC(wt) vs. Venus-bPAC(wt) |
|  |  |  | < 0.0001 | 2xLyn-Venus-bPAC(wt) vs. Venus-bPAC(wt) |
|  | **f** | ANOVA | 0.9035 |  |
|  |  | Dunnett’s multiple comparison | 0.9697 | R_M_ NT vs. R_M_ PACmn |
|  |  |  | 0.9412 | R_M_ NT vs. R_M_ bPAC(wt) |
|  |  | ANOVA | 0.9083 |  |
|  |  | Dunnett’s multiple comparison | 0.9049 | I_hold_ NT vs. I_hold_ PACmn |
|  |  |  | 0.8486 | I_hold_ NT vs. I_hold_ bPAC(wt) |
| **6** | **a** | ANOVA | < 0.0001 |  |
|  |  | Dunnett’s multiple comparison | < 0.0001 | bPAC(wt) vs. sensor only |
|  |  |  | 0.4400 | PACmn vs. sensor only |
|  |  |  | < 0.0001 | 2xLyn-Venus-bPAC(wt) vs. sensor only |
|  |  |  | < 0.0001 | Venus-bPAC(F198Y) vs. sensor only |
